# Chlorambucil-Loaded Graphene-Oxide-Based Nano-Vesicles for Cancer Therapy

**DOI:** 10.3390/pharmaceutics15020649

**Published:** 2023-02-15

**Authors:** Surabhi Kumari, Anuj Nehra, Kshitij Gupta, Anu Puri, Vinay Kumar, Krishna Pal Singh, Mukesh Kumar, Ashutosh Sharma

**Affiliations:** 1 Bio-Nanotechnology Research Laboratory, Biophysics Unit, College of Basic Science & Humanities, G.B. Pant University of Agriculture and Technology, Pantnagar 263145, Uttarakhand, India; 2 Department of Physics, Guru Jambheshwar University of Science and Technology, Hisar 125001, Haryana, India; 3 Basic Research Laboratory, Centre for Cancer Research, National Cancer Institute-Frederick, National Institute of Health, Post Office Box. Building 469, Room No. 216A, Frederick, MD 21702-1201, USA; 4 Department of Physics, CCS Haryana Agricultural University, Hisar 125004, Haryana, India; 5 Vice Chancellor Secretariat, Mahatma Jyotiba Phule Rohilkhand University, Bareilly 243006, Uttar Pradesh, India; 6 Department of Physics, Faculty of Science, Shree Guru Gobind Singh Tricentenary University, Gurgaon 122505, Haryana, India; 7 Department of Materials Science and Engineering, Ajou University, Suwon 16499, Republic of Korea

**Keywords:** graphene-oxide, chlorambucil, drug loading, nano-vesicles, cancer, in-vitro

## Abstract

In this study, the authors have designed biocompatible nano-vesicles using graphene oxide (GO) for the release of chlorambucil (CHL) drugs targeting cancerous cells. The GO sheets were first sulfonated and conjugated with folic acid (FA) molecules for controlled release and high loading efficiency of CHL. The chlorambucil (CHL) drug loading onto the functionalized GO surface was performed through π-π stacking and hydrophobic interactions with the aromatic planes of GO. The drug loading and “in vitro” release from the nano-vesicles at different pH were studied. The average particle size, absorption, and loading efficiency (%) of FA-conjugated GO sheets (CHL-GO) were observed to be 300 nm, 58%, and 77%, respectively. The drug release study at different pH (i.e., 7.4 and 5.5) showed a slight deceleration at pH 7.4 over pH 5.5. The amount of drug released was very small at pH 7.4 in the first hour which progressively increased to 24% after 8 h. The rate of drug release was faster at pH 5.5; initially, 16% to 27% in the first 3 h, and finally it reached 73% after 9 h. These observations indicate that the drug is released more rapidly at acidic pH with a larger amount of drug-loading ability. The rate of drug release from the CHL-loaded GO was 25% and 75% after 24 h. The biotoxicity study in terms of % cell viability of CHL-free and CHL-loaded GO against human cervical adenocarcinoma cell line was found to have lower cytotoxicity of CHL-loaded nano-vesicles (IC_50_ = 18 μM) as compared to CHL-free (IC_50_ = 8 μM). It is concluded that a high drug-loading efficiency and controlled release with excellent biotoxicity of CHL-GO offers an excellent application in the biomedical field.

## 1. Introduction

According to the World Health Organization report, 11 million people are diagnosed with cancer and 7 million people die from cancer every year. The deaths from cancer are the highest among those who die of disease worldwide [1]. The treatment of cancerous cells is limited to various chemotherapy, radiotherapy, photothermal therapy, and magnetic resonance imaging (MRI) owing to their inability to kill cancerous cells and protect healthy tissues inside the body [2,3,4,5,6]. A lack of specificity of available chemotherapeutic drugs toward cancerous cells often also causes severe damage to healthy tissues [7]. Thus, accurate delivery and release of drugs targeting cancer cells without any side effects such as baldness, bone marrow suppressions, etc., are desired [8,9]. Previously, conjugated drug delivery with cancer cell ligands has enabled the preparation of anticancer drugs effectively for targeting cancerous cells with minimum side effects to healthy tissues [10].

In today’s era, targeted drug delivery has revolutionized the field of cancer therapy [11,12]. Various mechanisms are utilized to target the drug to specific cancerous cells such as an antibody specific to the epitope on the cancerous cells [13]. Recently, folate receptors are being studied for targeting cancerous cells [14,15]. Folate binding protein, a glycosylphosphatidylinositol-anchored cell surface receptor for folate [16], has been known to be overexpressed in several human tumors including ovarian and breast cancers [17]. According to this literature, nanostructured drug delivery systems is permitted the development of unique platforms for the capable transport and controlled drug molecules release in the stiff microenvironment of diseased tissues of living cells, thus allowing an extensive range of functional nanomaterials/nanoplatforms for smart application in nanomedicine as well as bio-nanotechnology [14,18,19,20,21].

Nanomedicine and nano-delivery techniques are relatively newer but quickly developing science where materials at the nanoscale range are employed to serve as means of diagnostic tools or to deliver therapeutic agents to specifically targeted sites in a controlled manner [22,23]. Graphene is one of the newly emerging nanomaterials, which can be utilized in drug delivery. Graphene is an exciting material and has a large theoretical specific surface area, high intrinsic mobility, high Young’s modulus, thermal conductivity, optical transmittance, and good electrical conductivity [24,25,26,27,28,29,30,31,32]. As a result, graphene-based nanocomposites are now unique materials for formulations of micro-electrical devices, mechanic resonators, ultra-capacitors [33], nanosensors [34], and biomedicine [35,36]. Graphene-based sheets have also been tested as possible nanocarriers for delivering drugs [37] and also as functional biomaterials [38].

GO, first reported by Brodie in 1859, is another graphene-based material, being largely explored recently to be employed in drug delivery and biomedical applications due to its large surface-to-volume ratio, non-toxicity, and low cost. GO is associated with various ester, hydroxyl, and epoxide groups. These groups are utilized for the binding of groups which provides stability to GO under physiological conditions. Otherwise, GO will aggregate to form clusters at physiological conditions. Usually, researchers attached polyethylene glycol (PEG) polymer molecules (i.e., PEGylation) to GO to enhance its stability [22]. Recently studies have shown sulfonic group (derived from aryl diazonium salt of sulfanilic acid) can also be attached to GO to provide superior stability [39,40]. These modified nano-vesicles are loaded with various aromatic drugs. Doxorubicin and methotrexate were loaded on GO [22,41,42]. GO contains two aromatic planes, so two different aromatic drugs can be loaded onto two aromatic planes of GO. Doxorubicin and camptothecin were simultaneously loaded onto the surface of GO [39]. However, in drug delivery systems, agglomeration of GO sheets is a major problem which often results in poor delivery and loading efficiency. This could be avoided by the functionalization of GO sheets with a polymer group onto the surface of GO sheets followed by conjugation with a tumor-targeting agent to the delivery system.

Recent studies have shown that GO can be dispersed through functionalization, or chemically converted to make different graphene-based nanocomposites with excellent mechanical and thermal properties [25,26,29,30]. Similarly, some researchers have performed sulfonation of GO with the aryl diazonium salt of sulfanilic acid [39]. After that, the anti-cancer drug is loaded onto the exposed GO surface. GO has two aromatic planes and therefore, is capable of absorbing aromatic compounds, which suggests potential applications as drug carriers. The drug is bound to the GO by physisorption, mainly via π-π stacking and hydrophobic interactions [41]. To stabilize GO in physiological solutions and other biological media, grafting of PEG polymers is carried out onto the chemically activated surfaces and edges of GO [43]. It is observed that the pH of cancerous cells is slightly acidic as compared to normal cells. So, the pH-based release of anti-cancer drugs is an efficient method for drug release. Various researchers monitored the in-vitro pH-based release of anti-cancer drugs [44,45].

However, existing drug delivery systems have serious issues of segregation of GO sheets as well as weak drug delivery mechanism at active sites due to the lack of specific target agents. These issues could be rectified by surface functionalization (sulfonation) of the GO sheets followed by simultaneous conjugation of tumor target agent to the delivery system. The most widely used CHL drug is used against cancer cells of the lungs, head and breast tissues, skin, and ovary cells [46]. In the present work, a nano-vesicle covalently bound with FA is designed for targeted drug delivery in cancer treatment. The CHL was loaded onto the exposed surface of GO by the process of physisorption. This study comprises probably the first time loading CHL in the form of CHL-GO-FA onto GO (concentration is 1 mg.mL^−1^) and can potentially discover a safer mode of loading and targeting of this drug via GO, an organic and least toxic cheap nanomaterial. Further, the cell growth inhibition assay of GO-CHL and FA-GO-CHL against the human cervical adenocarcinoma cell line was conducted.

## 2. Materials and Methods

The various materials and methodology used in this work are described with sufficient details as follows.

### 2.1. Materials

Ethyl(dimethyl aminopropyl) carbodiimide (EDC), N-Hydroxysuccinimide (NHS), and graphite powder were purchased from Sigma-Aldrich, St. Louis, MO, USA. Sulfuric acid (H_2_SO_4_, assay NLT 97.0%), phosphorus pentoxide GR (P_2_O_5_ assay 98%), potassium permanganate (KMNO_4_, assay 99%), hydrogen peroxide (H_2_O_2_, 30%), and potassium persulfate GR (K_2_S_2_O_8_ assay 98.0%) were bought from Rankem, Molychem, Qualigens (Merck chemicals), and Loba Chemie, India respectively. All these chemicals were used in GO synthesis without further purification. Furthermore, CHL was purchased from United Pharmaceuticals, Rajasthan, India.

### 2.2. GO Synthesis

GO was synthesized by Hummers’ method [24,27,28,47]. About 3 mL concentrated sulfuric acid (H_2_SO_4_) was added to a mixture of graphite powder (2 g), potassium persulfate (1 g), and phosphorus pentoxide (1 g) in a 250 mL beaker. This mixture was kept at 80 °C for 6 h. Subsequently, this mixture was cooled down to room temperature and diluted with 200 mL of distilled water. This diluted mixture was filtered with Whatman’s filter paper and washed to remove the residual acid until the pH value of rinsed water became neutral. The obtained residue was dried at room temperature for 3 days. Then pre-oxidized graphite powder was obtained. This pre-oxidized graphite powder was subjected to oxidation using Hummers’ method [36]. Pre-oxidized graphite powder (1 g) was mixed with concentrated sulfuric acid (23 mL) at 8 °C. Then, potassium permanganate (3 g) was added gradually with constant stirring while keeping the mixture in the ice bath. After that, this mixture was kept at 35 °C for 2 h with continuous stirring followed by the addition of distilled water (47 mL). After 15 min, distilled water (14 mL) and 30% hydrogen peroxide (2.5 mL) were added to terminate the reaction. Later, this mixture was filtered and the residue was washed with diluted (10%) hydrochloric acid solution to remove metal ions. Then, this residue was washed and centrifuged (at 10,000 rpm for 10 min) repeatedly, until the pH of rinsed water reached neutral. Brown pasty material of GO was obtained, which was dried at room temperature for 3 days. The obtained GO powder (100 mg) was dissolved in 100 mL distilled water and bath sonicated for 8 h to obtain the GO solution (concentration = 1 mg.mL^−1^) [24,25,27,28].

### 2.3. Conversion of GO into GO-COOH

The ester, hydroxyl, and epoxide groups present in the GO sheets were converted into carboxyl groups to improve the stability of the graphene derivatives and to facilitate the chemical binding of biomolecules. For this, 10 mL GO solution (concentration of 1 mg.mL^−1^) was taken and 0.5 g sodium hydroxide and 0.5 g sodium monochloroacetate were added to it. After that, this solution was sonicated for 2 h to convert hydroxyl groups into carboxyl groups. Then, the obtained solution was neutralized with dilute hydrochloric acid. After neutralizing, the solution was repeatedly rinsed and centrifuged (1000 rpm for 10 min), until the solution was well dispersed in deionized water. For the removal of free ions, the solution was filled in a dialysis bag closed at both ends with cotton thread and tested for leakage. The dialysis bag was attached horizontally, fully stretched in a beaker containing 200 mL distilled water, and dialyzed for 48 h.

### 2.4. Sulfonation of GO

#### 2.4.1. Synthesis of Aryl Diazonium Salt of Sulfanilic Acid

Sulfanilic acid (200 mg) and sodium nitrite (80 mg) were dissolved in a 0.25% sodium hydroxide solution. Then, this solution was added dropwise to 0.1N hydrochloric acid in an ice bath to obtain aryl diazonium salt of sulfanilic acid.

#### 2.4.2. Sulfonation

A total of 4.5 mL aryl diazonium salt of sulfanilic acid was added to a 10 mL dispersion of GO, whose functional groups were earlier converted to carboxyl groups. Then, this mixture was kept in an ice bath for 2 h. After that, the mixture was filled in a dialysis bag closed at both ends with cotton thread and tested for leakage. The dialysis bag was attached horizontally, fully stretched in a beaker containing 200 mL distilled water, and dialyzed for 48 h. This obtained product was characterized by FT-IR. Then, this GO conjugated with a sulfonic group (GO-SO_3_H) was stored at 4 °C.

### 2.5. Conjugation of FA

NHS (N-Hydroxysuccinimide 18.25 mg) and EDC (Ethyl(dimethyl aminopropyl) carbodiimide, 12.5 mg) were added to 10 mL of GO-SO_3_H dispersion. Then, this mixture was sonicated through a water bath for 2 h. After that, 2 mL FA (0.5%, adjusted to pH 8 using sodium bicarbonate solution) was added to the mixture and it was stirred for 12 h. For the removal of unreacted materials, the mixture was filled in a dialysis bag closed at both ends with cotton thread and tested for leakage. The dialysis bag was attached horizontally, fully stretched in a beaker containing 200 mL sodium bicarbonate solution (pH 8), and dialyzed for 48 h. After that, the dialysis bag was transferred to a beaker containing 200 mL of distilled water, and dialysis was further continued for 12 h.

### 2.6. Characterization Studies

#### 2.6.1. Particle Size

The particle size distribution analysis was performed by a dynamic light scattering (DLS) measurement system (Microtrac, Microtrac-Retsch GmbH, Krefeld, Germany). The size measurements were performed five times and the result was averaged out for accuracy. The tests were performed at room temperature (25 °C). The powder sample was dissolved into a suspension of 5 sets of transparent disposable polystyrene cells.

#### 2.6.2. XRD Analysis

The crystal structure and phase analysis of GO were determined by X-ray Powder Diffraction (XRD) analysis. The XRD diffractometer was operated at a wavelength of 1.5404 Ǻ, with an operating voltage and current of 30 kV and 30 mA. The range of the Bragg angle used for powder diffraction was set within 2θ = 5–80° at a scan rate of 2°/min.

#### 2.6.3. Surface Morphology

The surface morphology of the dry GO and drug-loaded GO was observed by scanning electron microscopy (SEM, Hitachi 4800S, Tokyo, Japan) operated at 5 kV. The test samples were prepared by spraying the dispersed FA-GO nanocomposites onto a clean Petri dish followed by room-temperature drying and coating with a thin layer of Pd-Au for electrical continuity. To study the morphology features of GO at high resolution, transmission electron microscopy (TEM, Philips GT20, Cambridge, MA, USA) was also employed.

#### 2.6.4. Thermogravimetric Analysis

The thermogravimetric analysis (TGA) of GO powder was performed. The TGA thermogram was recorded using Perkin-Elmer Diamond TGA instrument (Perkin Elmer, Waltham, MA, USA). The scanning range of the TGA thermogram was fixed from 50–1000 °C.

#### 2.6.5. FTIR and UV-Visible

The presence of chemical bonding and functional groups onto GO was determined by Fourier transform infrared spectroscopy (FT-IR, model Nicolet 6700). The FTIR spectra were recorded at room temperature in the range of 500−4000 cm^−1^ using the KBr pellet method. The UV-Visible Spectrophotometer (Cary 100 UV-Vis) was also used to study the optical absorption characteristics of as-synthesized GO and FA-GO with and without drug loading in the range of 200–500 nm.

### 2.7. Loading of Drug

A total of 5 mL of FA-GO-SO_3_H aqueous suspension was taken and mixed with 5 mL CHL solution (1 mg.mL^−1^), and dissolved in distilled water. This solution was stirred for 24 h using a magnetic stirrer. Then, the free drug was removed by centrifugation. For this, GO loaded with the anti-cancer drug was taken in centrifuge tubes. The suspension was centrifuged at 4000 rpm at 4 °C for 30 min. The precipitate was then suspended in distilled water. Then, the absorbance of the supernatant was recorded at 264 nm to determine the amount of unloaded drug. The loading capacity of GO was evaluated after removing the free drug from the GO suspension. The standard curve of CHL was prepared at 264 nm. After centrifugation of GO, the supernatant was taken and the absorbance was measured at 264 nm. The concentration of the free drug was then measured using the standard curve. The loading efficiency and loading capacity were determined from the following equations [48].

(1) $$\mathrm{Amount}\;\mathrm{of}\;\mathrm{drug}-\mathrm{loaded}\;\mathrm{on}\;\mathrm{GO}=\frac{\mathrm{Total}\;\mathrm{drug}-\mathrm{Unloaded}\;\mathrm{drug}}{\mathrm{Amount}\;\mathrm{of}\;\mathrm{GO}}$$

(2) $$\mathrm{Loading}\;\mathrm{efficiency}=\frac{\mathrm{Total}\;\mathrm{drug}-\mathrm{Unloaded}\;\mathrm{drug}}{\mathrm{Total}\;\mathrm{drug}}\times 100\%$$

(3) $$\mathrm{Loading}\;\mathrm{capacity}=\frac{\mathrm{Total}\;\mathrm{drug}-\mathrm{Unloaded}\;\mathrm{drug}}{\mathrm{Amount}\;\mathrm{of}\;\mathrm{drug}\;\mathrm{loaded}\;\mathrm{on}\;\mathrm{GO}}\times 100\%$$

### 2.8. In Vitro Drug Release Study

Drug release from CHL-loaded GO was studied using a dialysis method. Dialysis bags were soaked before use in distilled water at room temperature for 12 h to remove the preservative, followed by rinsing thoroughly in distilled water. Two bags were prepared to contain GO loaded with CHL (5 mL). The bags were closed at both ends with cotton thread and tested for leakage. The dialysis bags were attached horizontally fully stretched in two beakers, one containing 80 mL of phosphate-buffered saline (PBS) at pH 7.4 and the other containing 80 mL of PBS (pH 5.5) as release medium. The bag was then fully immersed under the surface. The temperature was set at 32 ± 0.2 °C through a temperature-controlled centrifuge and the rotation speed of the shaker was set at 100 rpm. Higher temperatures than 42° may cause more sensitivity to drugs and damage the fresh cells. So, we kept this temperature below the normal body temperature [49]. Aliquots of the release medium were withdrawn for analysis at different time intervals. The absorbance of the collected sample was measured at 264 nm, the supernatant volume was replaced with fresh medium and then the sample was put back into the release medium. Release runs were continued for 24 h.

### 2.9. In Vitro Cytotoxicity Studies

#### 2.9.1. Maintenance of Cervical Cancer Cell Lines

The growth of the cells was induced in Dulbecco’s modified Eagle medium (DMEM) prepared in T25 Flasks using 10% (vol./vol.) heat-inactivated fetal bovine serum and 1% (vol./vol.) penicillin/streptomycin solution. The flasks were stored in an incubator at 5% CO_2_ atmosphere/37 °C. Further, 0.25% trypsin was added to the T25 flasks with cells for counting cells in the Neubauer chamber.

#### 2.9.2. Cell Growth Inhibition Assay

The cells were placed in a 96-well Falcon plate (Corning Costar, Corning, NY, USA) for 24 h. The assay was prepared from CHL-loaded FA-GO (CHL-GO) nanocomposites and drug-free FA-GO (drug-free GO) in a concentration range of 1–25 μM. After the attachment of the cells to the plate, 200 μL of fresh media was added to replace growth media followed by incubation of the resultant mixture for 24 h. When the incubation period was finished, 20 μL of 5 mg/mL MTT in PBS was added to each well and incubated again for 4 h. The MTT assay was solubilized by dissolving them in 200 μL of DMSO and shaking them for 6 min. The positive controls consisted of a culture medium with only cells. The absorbance of the cells was determined by a microplate reader at 264 nm. The cell viability (%) was calculated using the equation:

(4) $$\mathrm{Cell}\;\mathrm{viability}=\frac{\mathrm{Absorbance}\;\mathrm{of}\;\mathrm{sample}}{\mathrm{Absorbance}\;\mathrm{of}\;\mathrm{positive}\;\mathrm{control}}\times 100\%$$

## 3. Results and Discussion

### 3.1. Particle Size Analysis

The particle size of the drug delivery agents is the most important factor against tumor cells [50]. The tumor cells have a different vasculature than that of fresh body cells. Cancer cells are largely sized and are more heterogeneous in distribution, possess higher vascular density, and are more permeable [51]. The polydispersity nature of nanocomposites in our study would be beneficial in targeting a range of sizes and shapes of cancerous cells.

**Figure 1:**
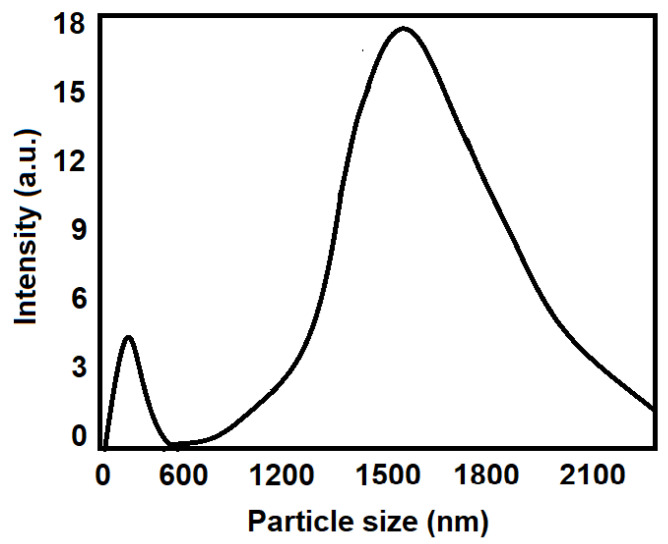
Particle size distribution of CHL-loaded GO nanocomposite in aqueous media.

The DLS measurements confirmed the swelling behavior of the FA-GO nanocomposite in an aqueous solution. The results showed a size distribution of 200–550 nm and 500–2500 nm. The distribution was bimodal as indicated by the dual intense peaks at 300 nm and 1450 nm respectively (Figure 1). In our study, the formation of smaller and average-sized nano-vesicles was confirmed from the DLS measurements and it could be due to the agglomeration of the nano-vesicles in an aqueous solution [52,53].

### 3.2. Surface Morphology

**Figure 2:**
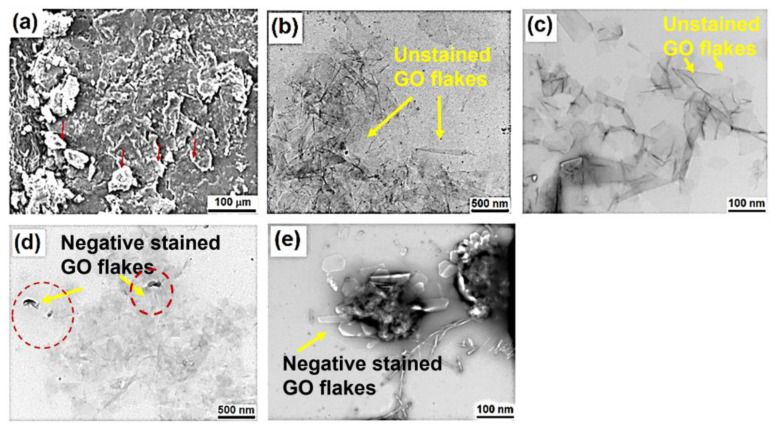
(**a**) SEM image of dried GO powder, (**b**,**c**) TEM image of unstained GO powder, and (**d**,**e**) TEM images of GO negatively stained loaded with CHL.

The surface morphology of the dried GO powder is shown by SEM image in Figure 2a. The GO powder shows aggregates and flakes due to the high surface energy layers of the graphene which segregate easily. The red arrows indicate the aggregate spots which consist of various GO layers. Further, to observe at high resolution, TEM images were analyzed to determine the size of GO flakes as shown in Figure 2b,c.

Figure 2b,c shows the TEM image of unstained GO. The well-defined faceted structure is seen for unstained GO at higher resolution. After CHL-loading onto GO, small porous and thick morphology on the GO plane is seen which shows a high loading of CHL. The swelling behavior of the GO with the drug is observed which is also earlier supported by the particle size distribution analysis (Figure 2d,e). This observation confirmed the formation of nano-vesicles of GO for the delivery of cancer drugs. Our results are consistent with those of the previous study of GO-based nanocarriers loaded with Paclitaxel drug [54].

### 3.3. Structural Analysis

**Figure 3:**
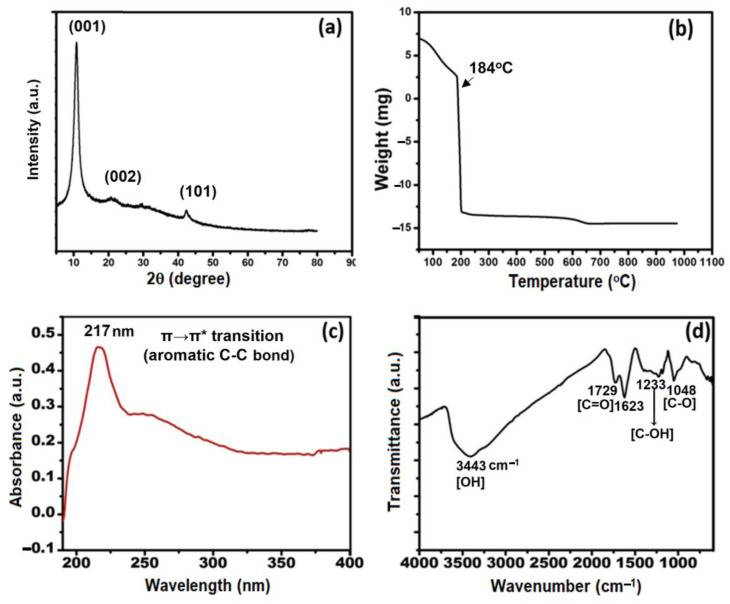
(**a**) XRD profile of dried GO powder, (**b**) TGA thermogram of dried GO from room temperature to 1000 °C, (**c**) UV-visible spectrum of GO (concentration = 1 mg.mL^−1^), and (**d**) FT-IR spectrum of GO.

Figure 3a shows the XRD diffraction pattern of dried GO powder. The prepared GO powder showed a reflection peak at 10.7° which is consistent with the previous studies [15,17,18]. A broad hump within the range of 2θ = 15°–30° has been designated to the reduced GO with a d-spacing of 0.37 nm. The stability of the GO powder at elevated temperatures is further verified by TGA analysis (Figure 3b). The TGA thermogram of GO shows that GO is stable until 50 °C. A severe weight loss increased with an increase in temperature beyond 50 °C up to 50 ± 2.3% weight loss occurred until 110 °C. A sudden weight loss occurred at 184 °C resulting in complete degradation of GO.

From the UV–vis spectra of GO (Figure 3c), a strong absorption peak at 217 nm was observed. This absorption peak is characteristic of exfoliated GO which corresponds to the π→π* transitions of the aromatic C=C bond [55]. The small hump at 300 nm in GO spectra corresponds to the n-π* transition of the carbonyl group [56].

The FTIR spectra of the GO showed a transmittance peak at 3443 cm^−1^ (O-H stretching vibrations), which was related to the OH groups. The other transmittance peaks at 1729 cm^−1^ and 1623 cm^−1^ (stretching vibrations from C=O), 1233 cm^−1^ (C-OH stretching vibrations), and 1048 cm^−1^ (C-O stretching vibrations) were typical of carbonyl and carboxyl groups as shown in Figure 3d [57].

### 3.4. Chemical Bonding and Functionalization of GO

**Figure 4:**
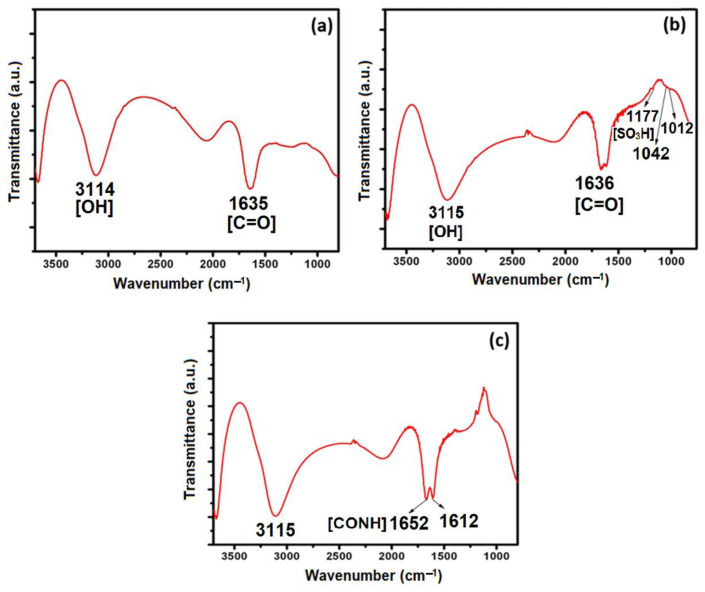
FT-IR spectrum of (**a**) GO-COOH, (**b**) GO-SO_3_H, and (**c**) FA-GO-SO_3_H.

The chemical bonding and functionalized groups were identified by the FTIR spectra as shown in Figure 4. The FT-IR spectrum of GO-COOH showed transmittance peaks at 3114 cm^−1^ (O-H stretching vibrations) and 1635 cm^−1^ (stretching vibrations from C=O). This spectrum of GO-COOH confirmed the conversion and synthesized GO-COOH had increased water solubility and more carboxylic acid groups for subsequent group attachment as shown in Figure 4a. The presence of SO_3_H groups in the GO was also confirmed by the FT-IR spectrum shown in Figure 4b. The transmittance peaks at 1177 cm^−1^ and 1042 cm^−1^ were arising from the vibration modes of the sulfonic acid groups. The transmittance peak at 1012 cm^−1^ was the characteristic vibration of a p-disubstituted phenyl group which is consistent with the previously published reports [58].

Conjugation of FA to GO-SO_3_H through the formation of an amide bond by the reaction between the NH_2_ groups of FA and COOH groups of GO-SO_3_H was confirmed by the FT-IR spectrum shown in Figure 4c. A new peak at 1652 cm^−1^ in the FT-IR spectrum indicated the presence of CO-NH groups in the FA-GO. The synthesized FA-GO dispersed in distilled water and physiological saline remained stable for several months, showing high stability.

### 3.5. Calibration Curve of CHL Suspension and Drug Loading

The absorbance of different concentrations of CHL was measured using a UV-visible spectrophotometer at 264 nm and the standard curve was plotted. The solvent used for the standard curve was distilled water. The drug-loading efficiency was studied with the help of a UV-visible standard curve. Using the standard curve (shown in Supplementary Figure S1) the different values of the released drug in various steps were calculated. After loading CHL onto GO, the suspension was centrifuged at 4000 rpm at 4 °C for 30 min to remove the free drug. Then, the supernatant was taken and its absorbance was measured at 264 nm. The absorbance of the supernatant was 0.058, the concentration corresponding to this absorbance was found to be 0.23 mg.mL^−1^. The total volume of the supernatant was 5 mL, so the amount of unloaded drug was 1.15 mg and the amount of loaded drug was 3.85 mg. The loading efficiency is equal to the concentration of the drug in the pellet divided by the total concentration of the drug in the formulations, as described by Equation (2). The total drug used during the preparation of GO-based nano-vesicle was 5 mL of 1 mg.mL^−1^ CHL. So using Equation (2), the loading efficiency of GO was found to be 77 ± 3.9%.

### 3.6. In Vitro Release Study

**Figure 5:**
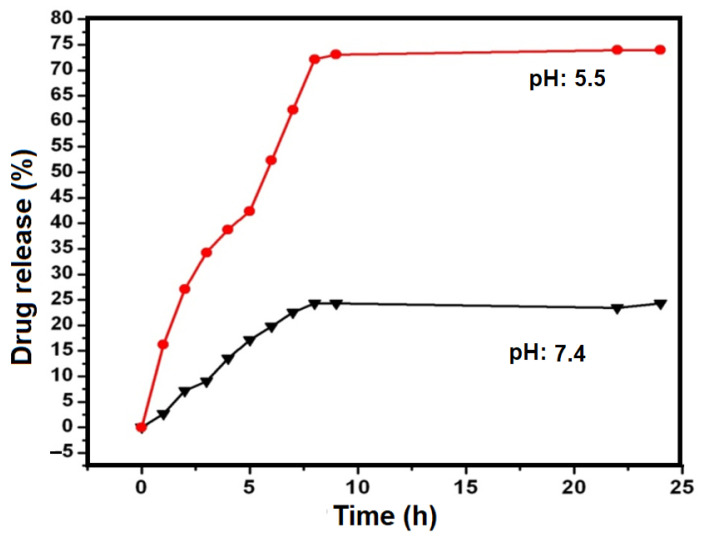
Drug release percentage of CHL-GO composite with time at different pH values.

In order to evaluate the controlled release potential of the formulations, the diffusion of CHL from the GO was investigated over 24 h. The drug release was determined by measuring the removal of CHL from the GO in phosphate-buffered saline (pH 5.5 and pH 7.4) at 37 °C. The aliquots were taken out after the interval of 1 h and the absorbance was measured at 264 nm for each sample. The experiment was continued for 24 h. In vitro, drug release mechanisms at pH 7.4 and pH 5.5 are shown in Figure 5.

The rate of drug released at pH 7.4 (black line, Figure 5) was quite slow as compared to the rate of drug released at pH 5.5 (red line, Figure 5). The drug released at pH 7.4 was much less at the first hour, about 2.7 ± 1.1%; it gradually increased to 24 ± 2.6% after 8 h. Moreover, the drug released at pH 5.5 was fast, and at the initial hours, 16 ± 1.4% to 27 ± 3.1% of the drug was released; it rapidly increased to about 40 ± 3.5% after 4 h, and finally, it reached 73 ± 3.9% after 9 h. Drug released at an acidic pH was faster because at low pH aromatic drugs (CHL) became more hydrophilic and water-soluble, thus leading to the release of more CHL. This release behavior is useful during the uptake of drugs by cancerous cells because cancerous cells uptake drugs by the process of endocytosis and package them in endosomes. Finally, these endosomes become lysosomes at an acidic pH. Inside lysosomes, the drug is released at a faster rate due to acidic pH. Additionally, the protonation of the amine group (NH_2_) of CHL led to the partial dissociation of hydrogen bonds between the drug and nano-vesicles. Moreover, a higher CHL loading was achieved due to the π-π stacking between the CHL and GO sheets which agrees well with the earlier studies with daunomycin and CNTs [59,60].

### 3.7. Cytotoxicity Studies

**Figure 6:**
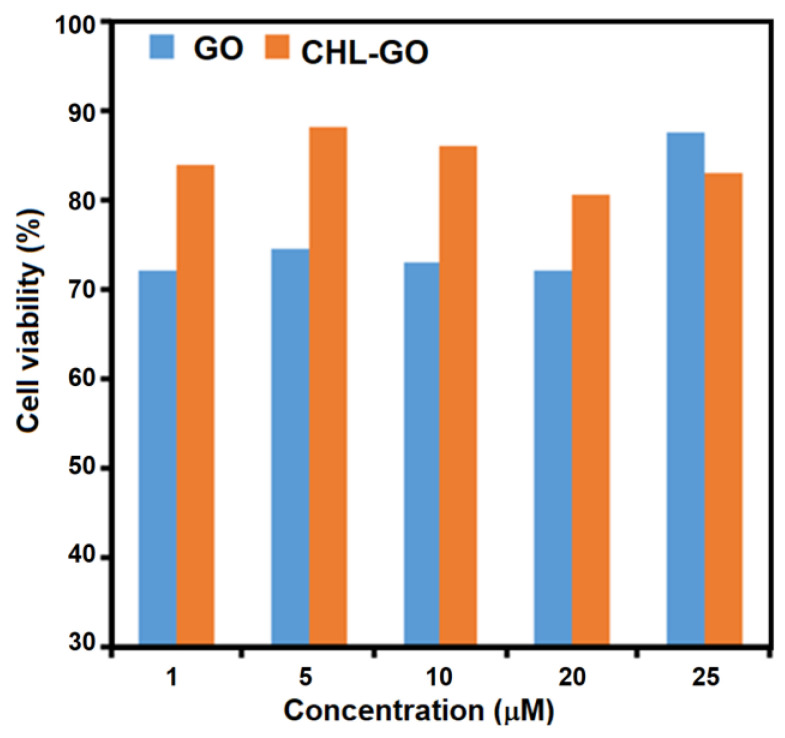
Cytotoxic study of GO and GO loaded with CHL.

To study the cellular effects of the synthesized GO and CHL-GO, the cytotoxicity test results were analyzed as shown in Figure 6. The results showed that lower concentrations of CHL-GO are highly effective on the viability of cells as compared to high concentrations of GO. Similar results were seen at different concentrations of GO-CHL.

Initially, gradual enhancement in the cytotoxicity was exhibited by GO and CHL-loaded GO. The mean values of cell viability for GO and GO loaded with CHL are 84.50 ± 4.67 and 71.34 ± 12.38, respectively. However, the cytotoxicity of CHL-GO containing up to 20 μM almost remained stable but the cytotoxicity of CHL-GO was lower at the same concentration. This phenomenon signifies that drug release from CHL-GO is a time-consuming and sustained release process. Sustained release of CHL from the nano-vesicles could be initiated through endocytosis; the nano-vesicles enter the cells and merge with lysosome-containing hydrolases enzymes [59,60]. Additionally, the conjugation of the CHL onto GO following π-π stacking and hydrogen bonding between GO and CHL also promotes sustained drug release. The viability of the cell lines was significantly decreased at all the CHL concentrations after 24 h. The inhibitory concentration values (IC_50_) also signify the low cytotoxicity of CHL-GO (IC_50_ = 18 μM of CHL-GO) as compared to drug-free GO (IC_50_ = 8 μM).

## 4. Conclusions

In this study, we have developed an FA-conjugated GO nano-vesicle for controlled drug delivery and release of the cancer drug CHL. The synthesized nano-vesicle is stable in a wide variety of physiological salines and easy to disperse in aqueous media. We have obtained a high drug-loading efficiency of CHL because of the high surface-to-volume ratio of GO planes and strong π-π stacking between CHL and GO. The drug release study from the GO was carried out at pH 7.4 and pH 5.5. The rate of drug released at pH 7.4 was quite slow as compared to the rate of drug released at pH 5.5. The drug released at pH 7.4 was very less at the first hour, about 2.7%, it gradually increased to 24% after 8 h. The drug released at pH 5.5 was fast; at initial hours, 16% to 27% of the drug was released. It rapidly increased to about 40% after 4 h, and finally, it reached 73% after 9 h. This pH-dependent drug-release behavior is useful during the uptake of drugs by cancerous cells. The cytotoxicity studies showed lower cytotoxicity of CHL-loaded nano-vesicles (IC_50_ = 8 μM) as compared to drug-free (IC_50_ = 8 μM). Thus, it may be concluded that CHL-loaded GO nano-vesicles provide controlled, targeted, and sustained release of drugs in cancer treatment.

## Data Availability

The data presented in this study are available on request from the corresponding author.

## Supplementary Materials

The following supporting information can be downloaded at: https://www.mdpi.com/article/10.3390/pharmaceutics15020649/s1, Figure S1: The calibration curve of CHL at 264 nm.

## Abbreviations

Graphene oxide (GO), folic acid (FA), chlorambucil (CHL), Fourier Transform Infrared spectroscopy (FT-IR), X-ray Powder Diffraction (XRD), Scanning Electron Microscope (SEM), Transmission Electron Microscopy (TEM), Energy-Dispersive X-ray Spectroscopy (EDS), phosphate-buffered saline (PBS), thermogravimetric analysis (TGA), etc.
