# Supplementary figures and images for: Chlorambucil-Loaded Graphene-Oxide-Based Nano-Vesicles for Cancer Therapy
Source: Pharmaceutics. 2023 Feb 15;15(2):649. doi: 10.3390/pharmaceutics15020649 (PMC9961782; doi:10.3390/pharmaceutics15020649)

## Supplementary Figures

Figure S1.

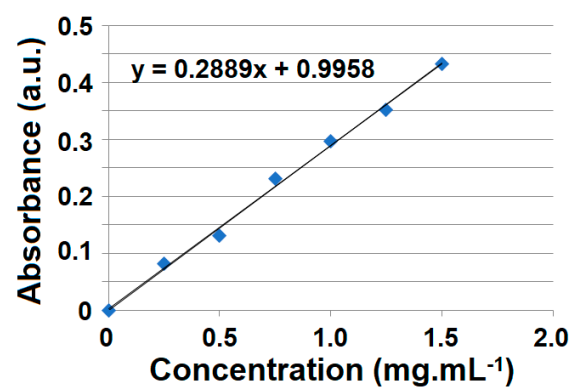

Figure S1. The calibration curve of CHL at 264 nm.

Supplement: Supplementary file 1 [file pharmaceutics-15-00649-s001.zip › pharmaceutics-2152973-supplementary.pdf]
